# “Descriptive Insight or Missed Accountability? Critical Methodological and Policy Gaps in a Study of Traditional Birth Attendants in Bangladesh”

**DOI:** 10.1002/hsr2.71837

**Published:** 2026-02-17

**Authors:** Ibadullah tahir, Hunain Shahbaz

**Affiliations:** ^1^ Ayub Medical College Abbottabad Pakistan

Dear Editor,

We found the qualitative study conducted by Mamun et al. on the knowledge, attitudes, and practice of Traditional Birth Attendants (TBAs) in Bangladesh [1] to be an interesting study due to the epidemiological significance of this domain; however, there were a number of methodological and conceptual limitations throughout this research that result in a significant reduction in the amount of evidence based material presented for developing global maternal and newborn health policies.

The small sample size (*n* = 11 TBAs) causes a lot of doubt on the amount of information and transferability from the findings. There is a claim of there being saturation; however, there is no clear explanation of how the researchers arrived at this conclusion of thematic redundancy across the range of sociocultural and geographic contexts in which they conducted their research [1]. Qualitative research standards clearly state the need for empirical evidence of saturation versus just assuming that saturation exists due to an increase in the number of focus groups that have been conducted thus far [2]. In addition, a lack of triangulation of the findings base off direct observations of women's viewpoints and skilled birth attendants, does not support any of the researchers' credibility in the review.

Additionally, the authors highlight numerous instances of the use of clinically harmful methods of managing the third stage of labour (manual surgical removal of the placenta) and managing newborns who do not breathe immediately after delivery (cord massage), use of mustard oil on newborns, and delaying transport to a higher‐level facility. However, the authors do not provide an adequately critical or evidence‐based assessment of the above stated interventions [1]. The aforementioned interventions are associated with multiple potential adverse outcomes (postpartum hemorrhage, neonatal hypothermia, sepsis, and asphyxia) that contribute significantly to the preventable death of infants [3, 4]. Describing them as mixed is inaccurate and leads to further normalization of non safe to already at‐risk populations.

Third, the study fails to adequately distinguish between individual knowledge deficiencies and systemic failures within the healthcare system. Structural limitations (e.g., inadequate access to trained birth attendants, lack of referral systems, and financial/geographic obstacles to using facilities for childbirth) are driving many of the practices of traditional birth attendants [5]. To focus predominantly on traditional birth attendant practices carries the danger of misdirecting policy makers to address only education while not paying enough attention to holding the healthcare system accountable.

Future research needs to use mixed methods designs through ethnographic observations and community input to gather information about how cultural differences and systemic influences vary across these areas of the world. Policy making should also focus on increasing SKBC, improving transportation, and reclassifying TBAs as referral agents. Unless the issues outlined above are addressed within a broad context, then the training‐based approach to improving maternal and infant mortality rates will likely have little effect.

## Author Contributions

**Ibadullah Tahir:** conceptualization, investigation, funding acquisition, writing – original draft. **Hunain Shahbaz:** validation, visualization, writing – review and editing. All authors have read and approved the final version of the manuscript.

## Funding

The authors received no specific funding for this work.

## Ethics Statement

The authors have nothing to report.

## Consent

The authors have nothing to report.

## Conflicts of Interest

The authors declare no conflicts of interest.

## Data Availability

The authors have nothing to report.
